# Surface-Mounted Bare and Packaged Fiber Bragg Grating Sensors for Measuring Rock Strain in Uniaxial Testing

**DOI:** 10.3390/s21092926

**Published:** 2021-04-22

**Authors:** Balarabe Wada Isah, Hisham Mohamad

**Affiliations:** 1 Civil and Environmental Engineering Department, Universiti Teknologi PETRONAS, Bandar Seri Iskandar Perak 32610, Malaysia; balarabe_16005697@utp.edu.my; 2 Department of Civil Engineering, Bayero University Kano, PMB 3011 Kano, Nigeria

**Keywords:** chained FBG sensor, measurement of deformation, uniaxial compression test, elastic behavior, strain-transfer coefficient

## Abstract

The paper explores the possibility of using high-resolution fiber Bragg grating (FBG) sensing technology for on-specimen strain measurement in the laboratory. The approach provides a means to assess the surface deformation of the specimen, both the axial and radial, through a chain of FBG sensor (C-FBG), in a basic setup of a uniaxial compression test. The method is cost-effective, straightforward and can be commercialized. Two C-FBG; one was applied directly to the sample (FBG_Bare_), and the other was packaged (FBG_Pack_) for ease of application. The approach measures the local strain with high-resolution and accuracy levels that match up to the existing local strain measuring sensors. The approach enables the evaluation of small-strain properties of the specimen intelligently. The finite element model analysis deployed has proven the adaptability of the technique for measuring material deformation. The adhesive thickness and packaging technique have been shown to influence the sensitivity of the FBG sensors. Owing to the relative ease and low-cost of instrumentation, the suggested method has a great potential to be routinely applied for elemental testing in the laboratory.

## 1. Introduction

The study of the mechanical behavior of rocks provides solutions to engineering problems related to a wide range of human activities, especially with the evolvement of large geotechnical engineering structures, such as deep tunnels, boreholes for oil and gas, and tunnels for storage of radioactive waste. Analyzing the mechanical behavior of rocks requires strain measurement. Most rocks are very stiff, and therefore their strain response to loading is minimal (microstrain) [1]. It requires a very accurate and high-resolution device to obtain a realistic stress-strain relationship of the rocks.

In the laboratory, the uniaxial compression test (UCT) is one of the basic tests routinely performed on rock specimens [2,3,4,5]. Both the International Society for Rock Mechanics (ISRM) [6] and the American Society for Testing and Materials (ASTM) [7] methodizes UCT. Over the years, devices like linear variable differential transformer (LVDT) [8,9], strain gauges [10,11], acoustic emission (AE) [9,12], digital image correlation (DIC) [13,14], and extensometer [15,16] were used during the UCT to determine the strain response of rocks with acceptable accuracy. High-resolution, accuracy, cost-effectiveness, simplicity, and reliability are the major factors that entail the selection of instruments for UCT. Difficulties in mounting, poor resolution, use of many cables, edgy acquisition, high-cost, and loss of alignment towards the end of the test by some devices, continued to exist, which is why other new methods continue to evolve.

Fiber Bragg grating (FBG) sensors have been adopted for measuring various quantities (strain, temperature, pressure, discharge, acceleration, force, displacement, vibration, etc.) as reflected in both application and review studies [17,18]. Perhaps due to the decreasing cost of FBG sensors and the advantages offered over conventional sensors, such as their embeddability, flexibility, small in size, immune to electrical or magnetic interference (EMI), resistance to corrosion, multiplexing, multifunctioning, high-resolution, and high measuring accuracy [17,19,20,21]. FBG sensor is usually imprinted on a bare fiber, and many points can be written along the same fiber forming a chain of FBG sensors. Sometimes FBG requires protection when they are used in a harsh environment. To enhance the mechanical strength and durability of FBG sensors, they are encapsulated in composite material or encased in a steel casing fabricated explicitly for a particular sensing purpose [19,22]. Many techniques of packaging FBG are reported in the literature based on a specific need [23,24,25].

The conventional strain gauges like electrical resistance strain gauges (SG) and linear variable differential transformers (LVDTs) get damaged when in contact with water, and each gauging unit requires many cables making it vulnerable to EMI and electrical noise, thereby affecting the measured strain. The typical extensometer demands to record values manually [26], which is hectic and tiring. Digital techniques entail moving the device frequently and require rigorous post-experimental computation, which is feverish and consumes time. Over the years, the FBG sensor has gained acceptance for use in determining the mechanical behavior of rocks [16,17,27,28,29,30]. FBG has been tentatively bonded to the core specimen for the determination of stiffness properties [16,17,27,28,29,30]. C-FBG can be used to measure both axial and radial strain of rocks. For simplicity and commercialization, C-FBG can be packaged as a single sensor for measuring both radial and axial.

The study intends to report the use of bare and packaged C-FBG sensors for axial and radial strain measurements during the UCT of rock. Two pairs of C-FBG sensors consisting of two packaged (FBG_Pack_) and two bare (FBG_Bare_), respectively and two strain gauges are bonded on each specimen. To ascertain the practicability and efficiency of packaged C-FBG, the strains of the FBG_pack_ are compared with that of the FBG_Bare_, strain gauge (SG) and linear variable differential transformer (LVDT). All the FBG sensors used in the experiments are of the same size and imprinted on the same type of optical fiber (OF) for consistency in the analysis. Numerical studies were conducted on the limestone geometric models to assess the strain transfer through the FBG_pack_ in comparison with the strain transfer through the FBG_Bare_.

## 2. Principle, Calibration and Packaging of FBG for Strain Sensing

### 2.1. Principle of FBG Strain Sensing Technology

Standard FBG sensors are usually inscribed on a single-mode optical fiber (SMF), which consists of a cylindrical inner core surrounded by cladding. The refractive index of the core is higher than that of the cladding. SMF allows the transmission of a single ray of light over a long-distance, making it suitable for various applications. The most common way of inscribing FBG is by using a phase mask method where a short segment of the fiber core is exposed to intense ultraviolet (UV) light through a medium (phase mask). When the UV light passes through the phase mask, it got diffracted to split into ±1 diffractive order. The fiber is positioned close to the phase mask, and once the fiber is exposed to the diffracted UV beams, it generates a periodic pattern to write a grating on the fiber core. Depending on the phase mask’s periodic pattern space, the grating period would be half of the phase mask’s period length. A reflection prism is used to inscribe FBG of various grating lengths with the aid of an adjustable linear guide to adjust the UV beam’s distance passing through the fiber core. The phase mask technique is simple and cheap. An inexpensive excimer laser can be used for writing FBG, which makes it suitable for the mass production environment.

Fiber exposure to UV light creates a permanent periodic alteration in the fiber core’s refractive index along the axis of the fiber called Bragg grating, which reflects a particular band of light wavelength and transmits all the others when broadband of light source is launched into the fiber. The UV pulse energy must be adjusted to a correct level to produce a perfect FBG sensor. The fiber coating must be removed with a standard stripping tool. The amount of alteration depends on the intensity of the UV light, photosensitivity of the SMF, and exposure duration. Usually, UV light is produced by a krypton fluoride (KrF) excimer laser at 248 nm or an argon fluoride (ArF) excimer laser at 193 nm [31].

**Figure 1:**
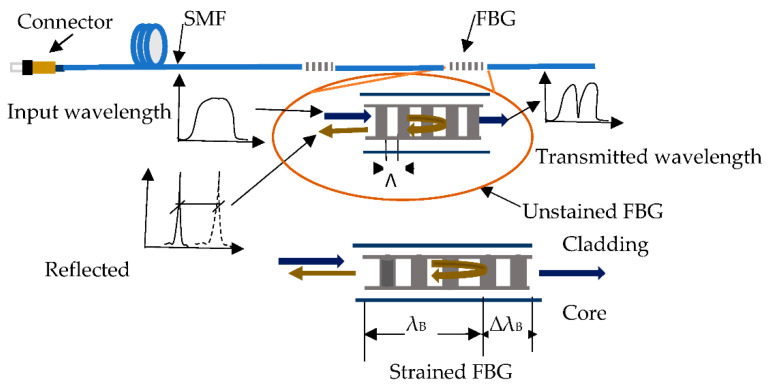
Working principle of fiber Bragg grating (FBG) sensors.

FBG sensing technology is well-known for measuring the strain and temperature of various structures. Figure 1 illustrates the working principle of the FBG strain sensor and how strain is measured with the FBG sensor. When OF is subjected to an external load, it alters the wavelength of the reflected light (Bragg wavelength), as shown in Figure 1. The relationship between Bragg wavelength and change in strain ∆ε or change in temperature ∆T can be determined with Equation (1) [32]. Temperature change also affects the Bragg wavelength shift through thermal expansion and contraction of the periodicity and refractive index of the gratings [33]. Therefore, it is important to apply temperature compensation where FBG is used in an environment where temperature varies.

∆λ_B_/λ_B_ = (1 − 𝑝*_eff_*)∆ε + (ξ + 𝛼)∆T (1)

where λ_B_ is the Bragg wavelength given as 2*n_eff_*Λ, 𝑝*_eff_*is the effective photo-elastic parameter related to the fiber core, ξ is thermal-optics coefficient of the OF core, 𝛼 is the thermal expansion coefficient of the OF, *n_eff_* is the refraction index of the core of the fiber and Λ is the grating period of index modulation. These parameters are constant for a particular fiber. When a measurement is taken at a relatively uniform temperature, any shift in the Bragg wavelength (∆λ_B_) can be determined using Equation (2). All the uniaxial compression experiments in this study were conducted under relatively the same laboratory temperature and in a relatively short period; therefore, the temperature effect is assumed negligible; hence, Equation (2) is used to obtain the FBG strain:

∆λ_B_ = λ_B_ε(1 − 𝑝*_eff_*) (2)

where (1 − 𝑝*_eff_*) is a strain constant given as *k*. 𝑝*_eff_*is constant (0.22); therefore, *k* is approximately 0.78 for a typical SMF.

### 2.2. FBG Strain Calibration

**Figure 2:**
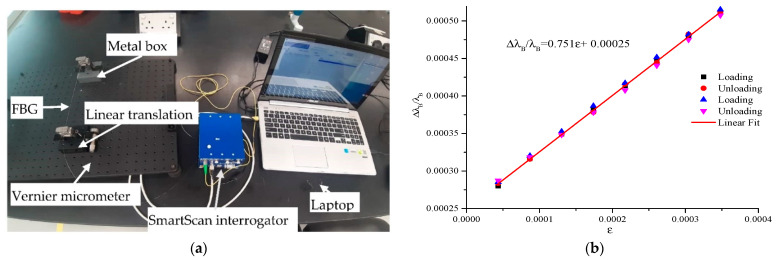
Strain calibration of FBG sensor. (**a**) FBG sensor strain calibration setup. (**b**) FGB strain calibration result.

Many researchers adopt the *k* value of 0.78, which is acceptable [20,34]. Nevertheless, some SMF may contain impurities; thus, it is good to determined *k* based on the available SMF. The authors have reported a laboratory technique of calibrating FBG sensors for strain measurement [35]. The apparatus consists of a linear translation stage fitted with a vernier micrometer, a fabricated box to aid the measurement, a mechanical vibration isolation platform, and an FBG fixed between the linear translation stage and the box. The FBG is connected to a smartfiber SmartScan interrogator, and it is used throughout the study. SmartScan is a very compact and robust interrogator. It is a wavelength division multiplexing (WDM) instrument based on a flexible, tunable laser source that enables high-resolution interrogation at multi kilohertz frequencies. The high-frequency scan rates allow oversampling and averaging to give an extraordinary resolution. SmartScan has four channels, scan frequency of 25 kHz, repeatability of less than 1 pm, a wavelength range of 40 nm from 1528 to 1568 nm and operation temperature between −15 to 55 °C [36]. The setup of the experiment is shown in Figure 2a. Both the translation stage and box are mounted on the platform at a distance of 230 mm apart. FBG sensor is fixed between the translation stage and the box. When the vernier micrometer is turned, the corresponding FBG reading is taken with the aid of interrogator. Loading and unloading values are taken at an interval of 0.01 mm. Figure 2b presents the calibration result. The *k* value was found to be 0.751, with R^2^ of 0.99 and it is used to determine FBG strain for this study.

Both FBG and SG measure strain by attaching on an object with glue. FBG has high-resolution and more robust over a long-range than SG. FBG sensors can keep pace with SG price-wise. They can even be written in the laboratory on SMF, which is very cheap. If the inscribing machine is available, skilled personnel can imprint FBG on a considerably cheap OF. FBG sensors provide surpassing qualities, making them suitable for specific specialized applications. For instance, FBG sensors work very well with composite materials. They are ideal for measuring high strain (>10,000 μm/m).

### 2.3. Preparation, Packaging and Attachment of C-FBG Strain Sensors

Several types of fibers can be used to package FBG sensors for engineering purposes: carbon fiber-reinforced polymer (CFRP), glass fiber-reinforced polymer (GFRP), synthetic fiber-reinforced polymer (SFRP). The CFRP was selected due to its strength and lightweight. In addition, the modulus of CFRP and SMF cable is approximately the same. This study harnessed the quasi-distributed sensing capability to package the FBG sensor that can measure both the radial and axial strain of rocks. The packaging consists of a layer of CFRP with FBGs attached to it using cyanoacrylate adhesive followed by a layer of Sellotape to add more protection. The packaging and attachment of all sensors were conducted using cyanoacrylate adhesive throughout the study as it was found to be suitable for pasting FBG on rocks [37].

**Figure 3:**
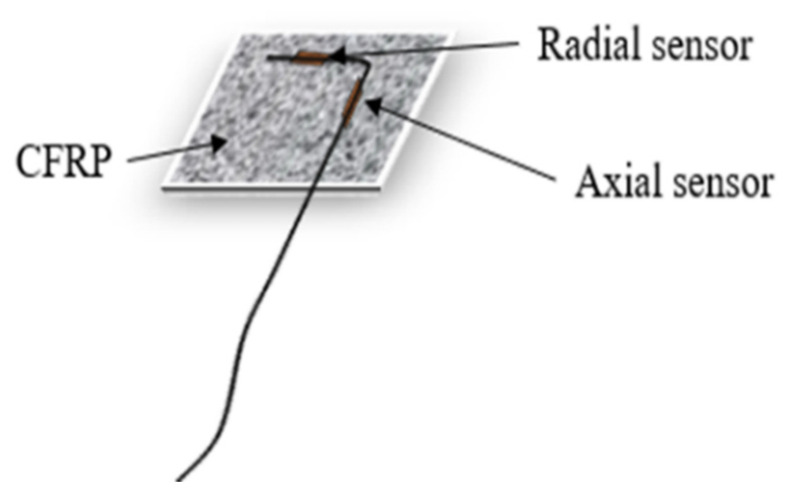
FBGs packaged for axial and lateral strain measurement.

A pair of FBG sensors are inscribed 25 mm apart, and each pair was spliced to a connector to enable data login. An L-shaped CFRP of approximately 20 mm by 30 mm was prepared, and each pair of FBG sensors was attached to the CFRP of 0.5 mm approximate thickness. Cyanoacrylate adhesive was adopted for pasting the coupled FBG sensors on the CFRP. The coupled FBG sensors were pasted to the CFRP with cyanoacrylate adhesive. They were packaged such that one is oriented to measure the strain *Y*-axis and the other-oriented to measure deformation along *X*-axis (Figure 2). By doing so, both axial and radial strain can be measured with a single FBG_Pack_. To enable data comparison, another pair of multiplexed FBGs (FBG_Bare_) were also prepared and spliced to OF connector. Figure 3 illuminates the pictorial view of the final FBG_Pack_ sensor.

**Figure 4:**
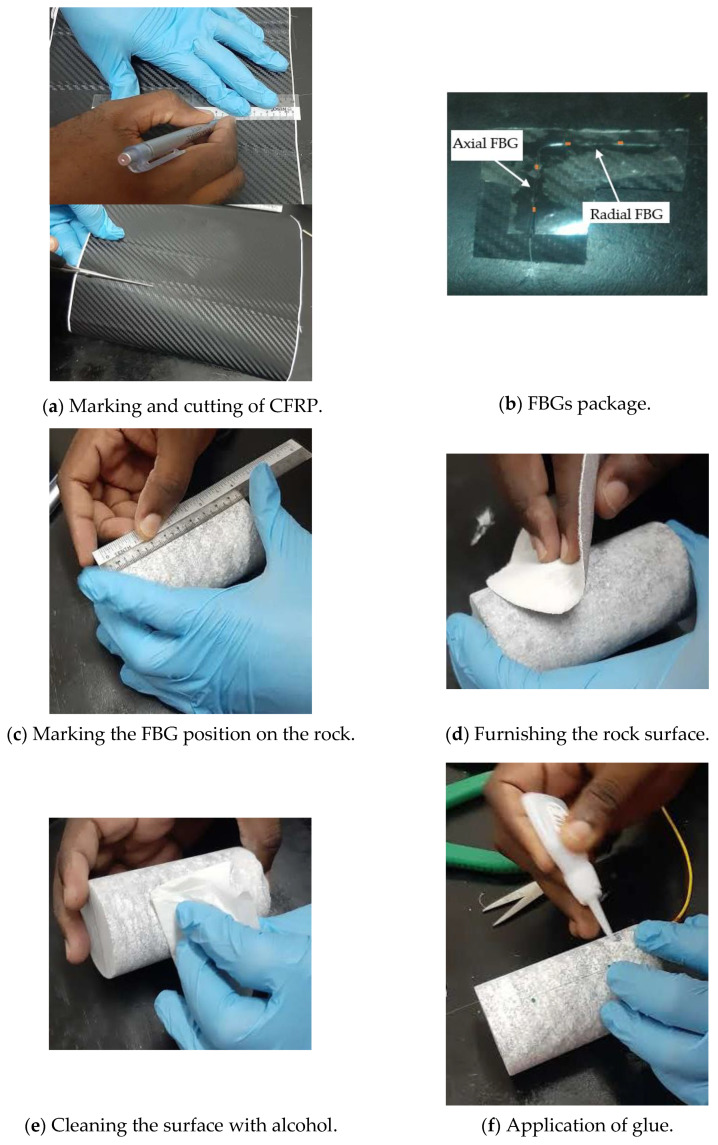

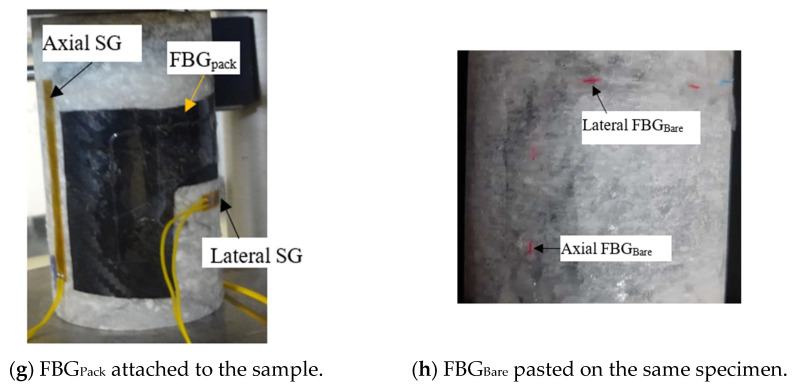
The process of packaging and pasting of both FBG_pack_ and FBG_Bare_ on limestone rock.

To minimize attenuation resulting from bending or medium of transmission, standard SMF (SMF-28e) was adopted in all experiments for imprinting FBG sensors and connecting the sensors to the interrogator. In each C-FBG sensor, two FBG sensors are inscribed on the same SMF, with each grating assigned a different wavelength to enable the interrogator to capture and record all readings simultaneously using wavelength division multiplexing. All the assigned wavelengths in this study are within the range of 1528 to 1560 nm (SmartScan wavelength range) to ensure that each sensor works within a particular spectral range. The FBG sensors were supplied by NanZee Sensing Technology Ltd. and were inscribed using the phase mask method described in Section 2. Table 1 shows the typical specifications of the FBG sensors used in the study. All the FBG_Pack_ were prepared at least two days prior to testing and attached to the rock specimen a day before the testing. The sensor position was marked on each sample, and the surface was cleaned with alcohol to provide a clean surface free from dusty and oily particles that can affect the bonding strength. Figure 4 shows the steps for sensor preparation, packaging and attachment to rock specimen. FBG_Bare_ was also attached directly to each sample for measuring lateral and axial strain. The FBG_Bare_ provides a basis for comparing the performance of the FBG_Pack_. Moreover, SG and LVDT were also deployed for validating the results obtained from FBG sensors. The FBG_Pack_ is fixed to the required position on the host rock with a thin layer of cyanoacrylate.

## 3. Materials and Methods

In this study, a limestone rock core of 50 mm in diameter was drilled from an underground sewerage pipeline installation site in Ipoh, Perak, Eastern part of Malaysia. The core was then cut to the size of a laboratory testing specimen of approximately 2.0 aspect ratio (length/diameter). The two faces of the rock are trimmed cautiously to obtain a perfect right circular cylinder following the International Society for Rock Mechanics (ISRM) standard. The specimens are oven-dried for 24 h before testing to ensure testing on the dry state.

**Figure 5:**
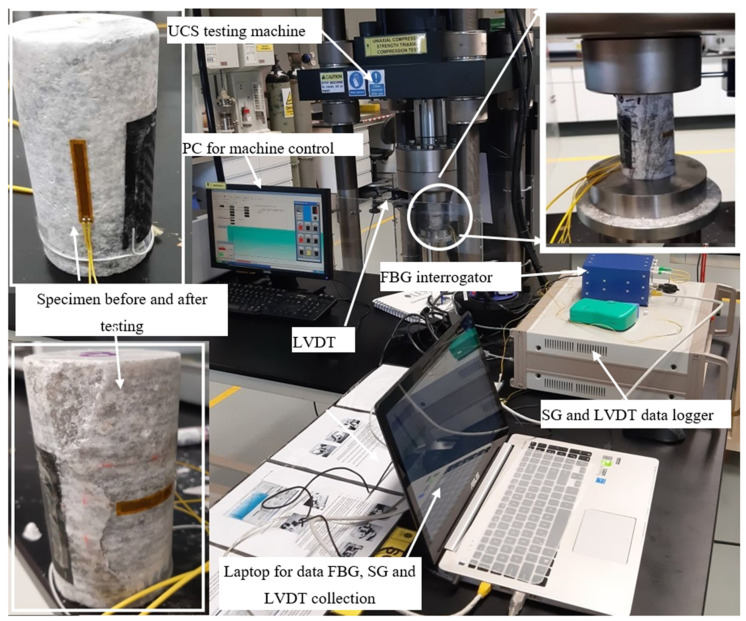
Experimental setup.

The uniaxial compression test is conducted with a servo-controlled RT-1000 compression test machine manufactured by an IPC global rock tester that has an axial loading capacity of 1000 kN. The testing machine is controlled with the aid of software installed in a computer that enables the user to set parameters according to the testing condition. The software provides the user with several options, including the type of the test and the loading condition (axial force-controlled or displacement controlled). Limestone core instrumented with FBG_Pack_, FBG_Bare_, and SG attached to the specimen along both axial and radial direction to record axial and radial strain, respectively, is placed on the lower plate of the machine. For a better comparison of the recorded data, the FBG_Pack_, FBG_Bare_, and SG are positioned adjacent to each other, respectively. The uniaxial test was carried out using a displacement-controlled rate of 0.01 mm/s. Additionally, an LVDT with a measuring range of 10 mm was mounted on the machine frame with the tip touching the top-loading plate to record the axial displacement. Figure 5 shows the testing setup, including how the SG and FBG data are recorded during testing.

Uniaxial testing on the limestone was carried out per the ISRM guidelines and ASTM D7012-07 standards [6], and the test took 2–5 min to complete. The FBG sensors data were recorded throughout the testing period at a frequency of 1000 Hz. FBG measures wavelength, which is converted to strain using the calibration factor obtained in Section 2.2. The converted strains were used to plot the FBG stress-strain curves for each tested specimen. The SG and LVDT data were recorded using an 8 channels dynamic and static strain measurement data logger (GTDL-160). The SG, LVDT and data logger are manufactured by JooShin corporation Korea [38]. The FBG data were recorded with the aid of a SmartScan software installed in a laptop, while the SG and LVDT data were recorded with the support of multiscan software that comes with GTDL-160 data logger (Figure 5).

## 4. Results and Discussion

### 4.1. Laboratory Experiment

This section presents and discusses the results of the uniaxial compression test experiments performed on limestone specimens equipped with C-FBG sensors (FBG_Bare_ and FBG_Pack_), SG, and LVDT. FBG_Bare_, FBG_Pack,_ and SG are attached to the sample for measuring on-specimen (Local) strain, while LVDT is attached to the upper loading plate to measure the crosshead deformation. The experiments were conducted at relatively room temperature, ignoring the effect of changes in temperature on FBG sensors. The C-FBG sensors were connected to two channels of the SmartScan interrogator for data logging. The SG and LVDT were connected to the same data logger, and both were controlled with the same software installed on a laptop. All the sensors were tested by logging in a few seconds of data to ensure all software and hardware are working correctly before the actual testing begins.

**Figure 6:**
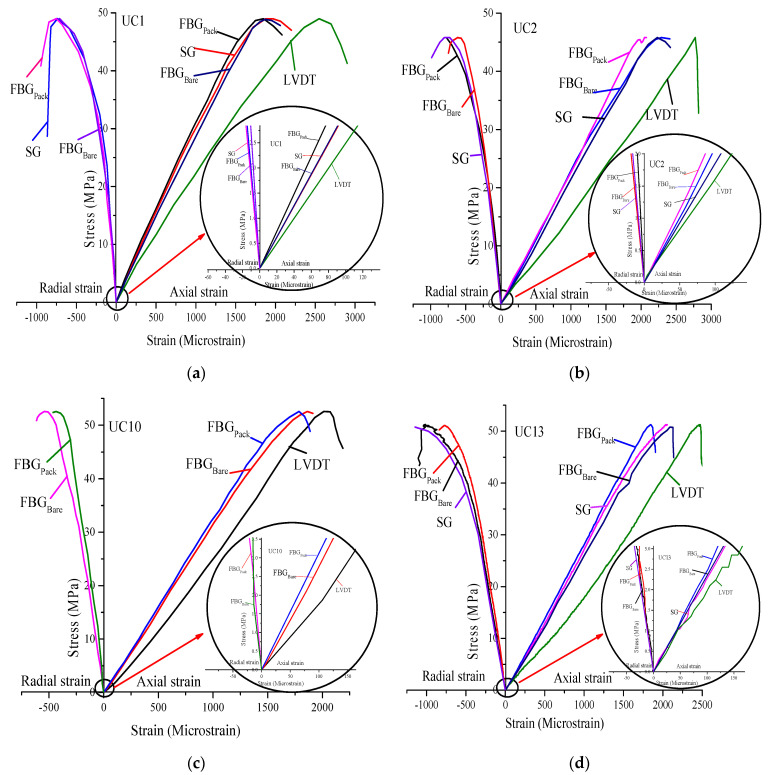

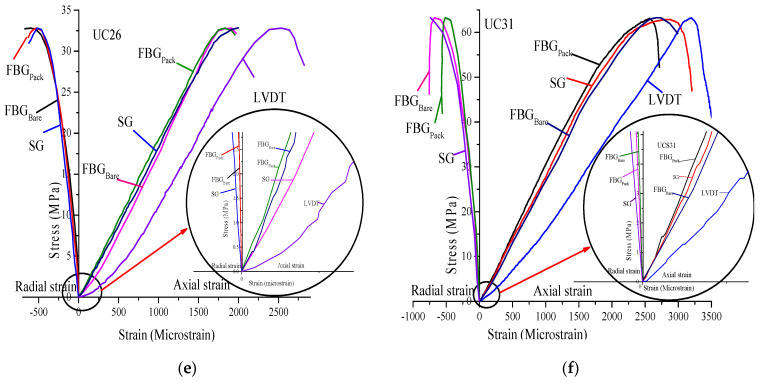
Experimental stress-strain curves of limestone specimens (**a**) UC1, (**b**) UC2, (**c**) UC10, (**d**) UC13, (**e)** UC26, and (**f**) UC31.

The stress was plotted against the axial and radial strain response recorded by FBG_Bare_, FBG_Pack_, SG, and LVDT. A total of 6 specimens were tested, readings from all sensors were recorded, and the results are presented in Figure 6, with each response designated by a distinct color.

Homogenous specimens were selected by visual inspection. As indicated in Figure 6, there is a consistency in the stress-strain curves of all the sensors plotted and for all the specimens. Slight variations in strain response of the local sensors (FBG_Bare_, FBG_Pack_, and SG) are observed in all the specimens. The LVDT data recorded a much higher strain response in all the samples. The higher strain recorded by LVDT is evident as it measures the accumulated deformation from the bottom of the load cell to the bottom plate, while the FBG_Bare_, FBG_Pack_, and SG measure the strain on the specimen. The findings are in agreement with [8,13,39,40], which pointed out that measuring the deformation of the sample using techniques other than the local deformation measuring techniques is accompanied by bedding and system compliance error. During the experiment, two significant damages occurred on the rocks; axial splitting with soft crackling and multi fracturing accompanied by loud crackling and rapid unloading to zero, which agrees with [41,42].

One of the crucial requirements of a local strain measuring device is high-resolution and the ability to capture the small strain response of rock since the rock response to deformation is generally small [40]. To understand the initial strain state achieved by the sensors, the first few loading stages are magnified, as shown in Figure 6 for each tested specimen to appreciate the performance of FBG sensors. Both the FBG_Bare_ and FBG_Pack_ have demonstrated remarkable performance more than LVDT and similar to the SG in all the specimens.

**Figure 7:**
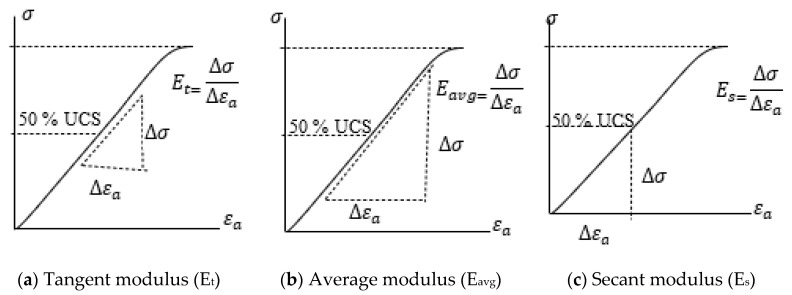
The method used to determined Young’s modulus at 50% UCS [7].

Furthermore, elastic parameters (Young’s modulus and Poison’s ratio) that provide vital information required for the design of excavation, borehole stability, and defining parameters needed for constitutive models, etc., are computed from the stress–strain curves. All the moduli are determined at about 50% of UCS. Tangent modulus (*E_t_*), average *E_avg_* modulus, secant modulus (*E_s_*), and Poisson’s ratio (*v*) are computed as per ASTM D 7012-04 [43]. Figure 7 demonstrates the method adopted for computing Young’s modulus, while the Poisson’s ratio is calculated using Equation (3).

*v =* (*E_t_* of axial curve/*E_t_* of lateral curve) (3)

The UCS and variations in the computed Young’s modulus of all the specimens are shown in Table 2. While Young’s modulus obtained from FBG_Pack_ is slightly higher in all the samples, the modulus obtained from FBG_Bare_ is almost the same as that of the SG in all cases. For instance, considering specimen UC1, with reference to SG, *E_t_* for FBG_Pack_, is 8% higher, for FBG_Bare_ is 1.44% lower, and for LVDT is 26.9% lower.

Moreover, a parameter *m* based on Equation (4) was computed, and the results are tabulated in Table 3:

*m* = *E_tsensor_*/*E_tSG_* (4)

In Equation (4), *E_tSG_* and *E_tsensor_* represent the tangent modulus obtained from the SG and each of the other sensors (FBG_Bare_, FBG_Pack,_ and LVDT), respectively. *m* measures the relativeness of the C-FBG sensors and LVDT readings to that of the SG. Values close to unity indicate perfect agreement between the stiffness obtained from SG and other sensors. The values of *m* from all the C-FBG sensors approach unity, which means a definite correlation between the stiffness from SG and both C-FBG sensors. The values of *m* for the LVDT are further away from unity; hence, considerable variation in the stiffness, and it even proves the accumulation of bedding and machine compliance error associated with measuring deformation on the machine load cell.

Table 4 summarizes the calculated Poisson’s ratios (*v*) of the rock from all the sensors. The calculated *v* from FBG_Pack_ is higher in most specimen than the FBG_Bare_ and SG, except for the samples UC2 and UC13 that have the same values of *v* as SG. There is a close resemblance in all the deformation parameters of FBG_Pack_ and FBG_Bare_ with SG.

### 4.2. Finite Element Modeling (FEM)

FEM was conducted with Ansys software version R3 2019 to investigate the Strain-Transfer Coefficient (STC) between the rock surface and the FBG in both the bare and packaged FBG sensors (Equation (5) defines STC). The geometric models were constructed in Solidworks for simplicity and then imported to Ansys R3 for modelling. The geometry of the model was made up of 5 parts; the rock specimen, adhesive, bare or packaged FBG sensor, top, and base plates. The rock was designed as a solid deformable cylindrical element of the same size as the experimental test specimen (100 mm by 50 mm); the top and base plates were designated as circular steel plates of 60 mm diameter and 20 mm length. The mesh was a structured mesh with 3D hexahedral elements. The method of meshing used was multizone. Acceptable mech quality was attained with 63,744 total elements and 267,002 nodes. The mesh size of plates and rock were 5 and 1 mm whereas the size of adhesive, packaging and FBG mesh was 0.5 mm. The contact between the specimen and both the top and base steel plates was set as fictional with a 0.02 frictional coefficient. All the remaining contacts were kept bonded. The loading (10 steps) was assigned as a displacement control from the top plate, portraying the experimental condition. Table 5 shows the properties of all the materials used in the FEM. The properties of CFRP and cyano were obtained from the manufacturers [44,45]. The parameters of the UC31 specimen were adopted for the FEM analysis.

(5) $$STC=\left(1-\frac{\varepsilon_{r-\;}\varepsilon_{f}}{\varepsilon_{r}}\right)\times 100\;\left(\%\right)$$

where *ε_r_* and *ε_f_* represent deformation on rock and FBG.

**Figure 8:**
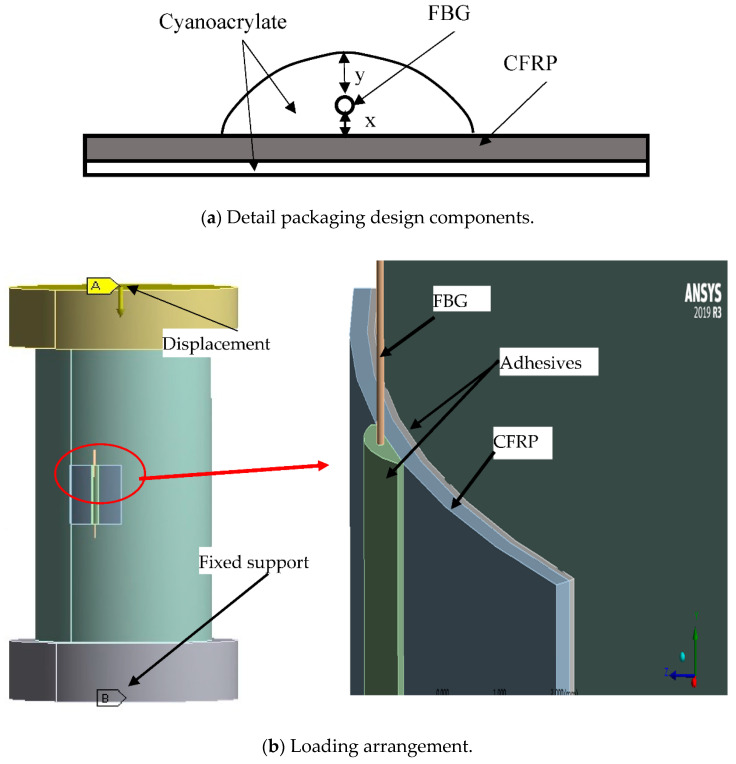

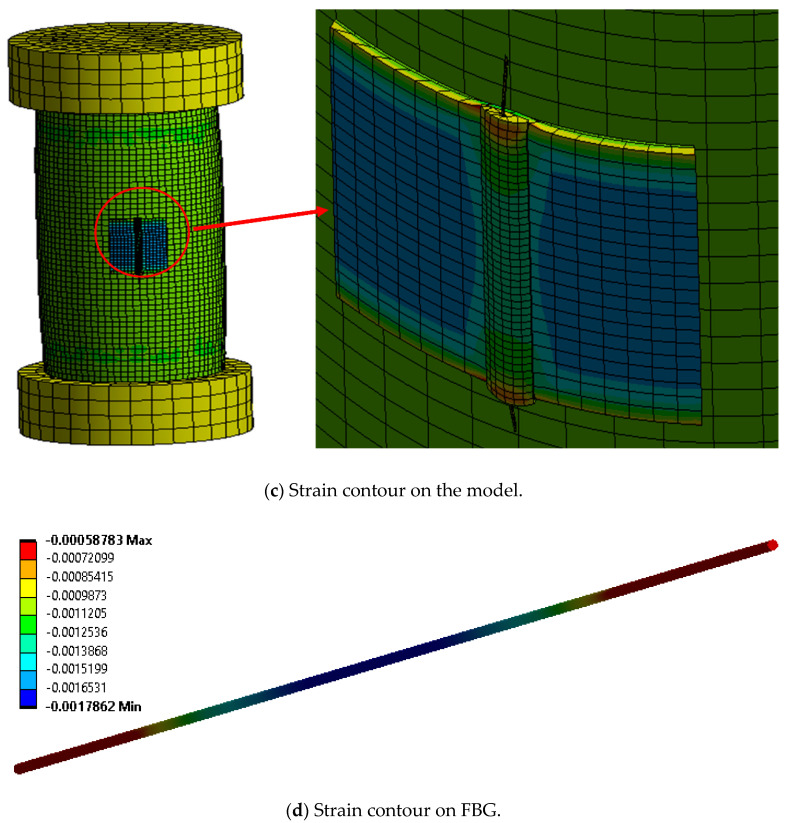
Finite element modeling (FEM) analysis.

The FBG packaging was designed as shown in Figure 8a. The shape of the adhesive on top of the CFRP was presumed to be elliptical, having 2 mm width, and the FBG was immersed in the adhesive. The top adhesive thickness is designated as y, while the bottom adhesive thickness is x. The thickness of CFRP and the adhesive that attached the package sensor on the rock were kept at 0.3 and 0.1 mm, respectively. During the FEM, displacement was applied at the top plate incrementally, and the base plate was kept as fixed support. The model elements, meshing, loading, and FEM analysis are shown in Figure 8.

An STC analysis of the package FBG sensors was carried out as per Table 6. The analysis was in three classes; in class 1, 2, and 3, the package length was kept at 15, 20 and 30 mm, respectively, while the bottom adhesive thickness (x) was varied, keeping the adhesive thickness on top of the FBG (y) constant. Studies have shown that the effect of the top thickness (y) is negligible [46,47]. As such, it was kept at 0.2 mm throughout this study.

**Figure 9:**
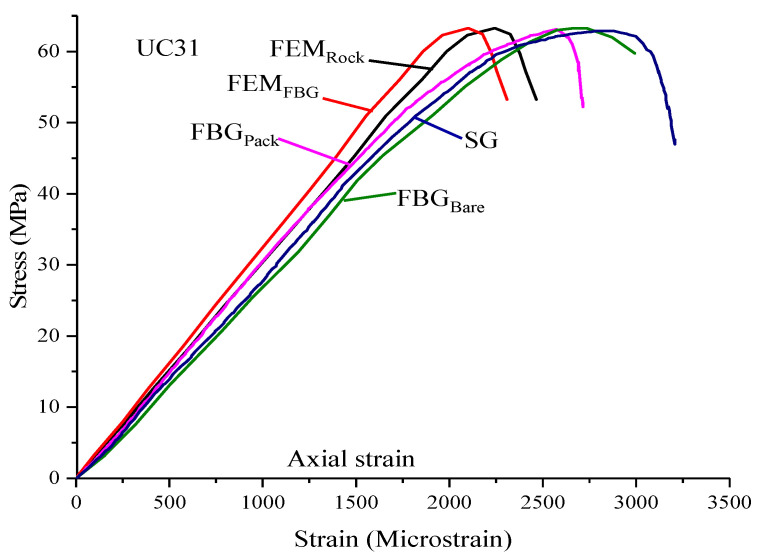
Comparison of experimental and numerical results of sample UCS31.

The comparison of the stress-strain curves of FBG_Bare,_ FBG_Pack_, strain from FBG and rock elements of FBG_Pack_ FEM are shown in Figure 9. The stress-strain curves of FBG_Pack_ FEM is based on a packaging length of 30 mm, the bottom thickness of 0.05 mm and 0.05 mm thickness of adhesive between the CFRP and limestone. All the curves are typical of brittle character; hence, both models were suitable for characterizing the behavior of limestone rock tested. There was an agreement between the resulted of the tested and FEM specimen, which validated the suitability of FBG sensors for strain measurement in uniaxial compression testing. Values of *E* were computed as illuminated in Table 7. Even the stiffness parameters of tested and FEM specimens accorded well with each other. Though all the results were in agreement with the limit of experimental error, FBG_Pack_ results were more similar to FEM than FBG_Bare_.

Table 8 presents the effect of packaging length and bottom adhesive thickness (x) on STC. It can be observed that the length of the package sensor influenced the STC. When the length increased, the STC also increased. There was also a reduction in STC with an increase in the bottom thickness. STC was more than 90% at 30 mm packaging length in all adhesive thickness. Both the length and bottom thickness had influenced the STC. The highest value of STC was observed at 30 mm packaging length and 0.05 mm bottom thickness (x); therefore, it was selected for all subsequent analyses. Adhesive thickness was shown to have greatly influenced STC.

**Figure 10:**
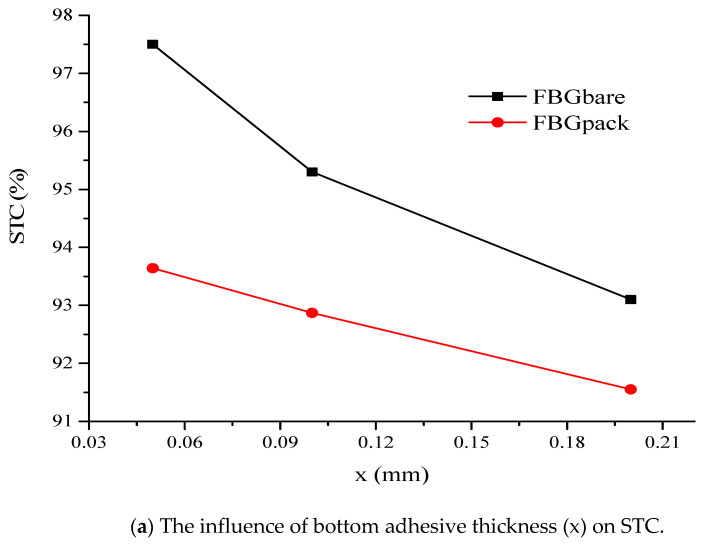

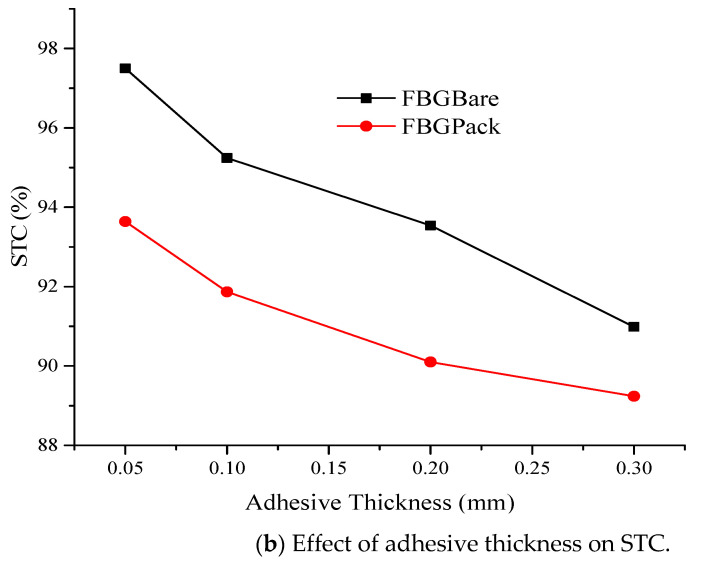
Sensitivity analysis of FBG_Bare_ and FBG_Pack_.

Furthermore, the comparison of STC between the FBG_Bare_ and FBG_Pack_ demonstrated that FBG_Bare_ was more sensitive than FBG_Pack_ (Figure 10). This may be attributed to the presence of the packaging layer in the case of FBG_Pack_. Figure 10a compare FBG_Bare_ and FBG_Pack_ based on the variation of the bottom thickness (x), while the effect of varying the adhesive thickness between FBG_Bare_ and limestone, CFRP and limestone are shown in Figure 10b, respectively. It is important to note that the bottom thickness was the same as the thickness between the FBG and limestone for FBG_Bare_. STC reduced with the increase in adhesive thickness.

**Figure 11:**
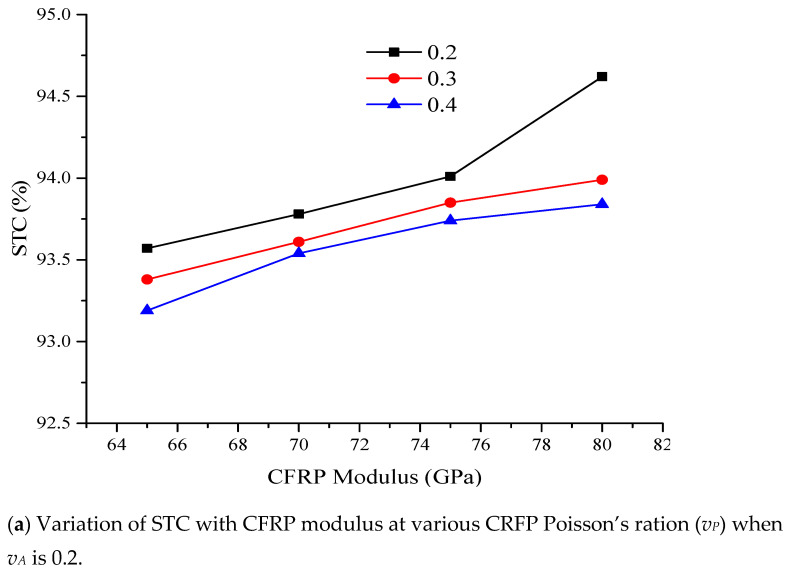

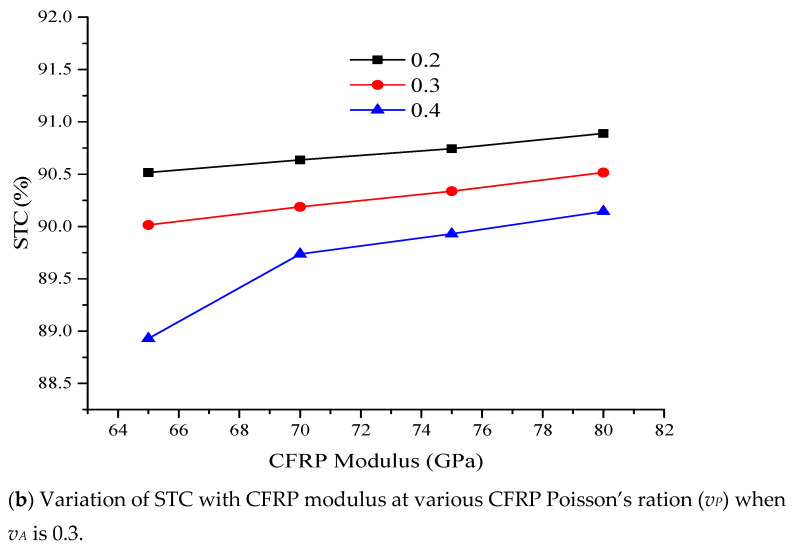
Influence of packaging modulus and Poisson’s ratio on STC.

Figure 11 shows the variation of STC as a function of packaging modulus and Poisson’s ratio (*v_P_*) at different values of adhesive Poisson’s ratio (*v_A_*). In Figure 11a, *v_P_* varied as 0.2, 0.3, and 0.4, CFRP modulus varied as 65, 70, 75 and 80 GPa, while *v_A_* was kept constant (0.2). The same scenario was repeated for *v_A_* of 0.3. The sensitivity of the packaged FBG sensor rose with increasing CFRP modulus. STC declined with the rise in both *v_A_* and *v_P_*. This implies that the increase in the package modulus courses corresponding increase in the sensitivity of the sensor, while a rise in Poisson’s ratio of both adhesive and packaging material reduced the sensitivity of the sensor.

### 4.3. Study of the Effect of Frictional Coefficient (FC) between Steel Plates and Specimen

**Figure 12:**
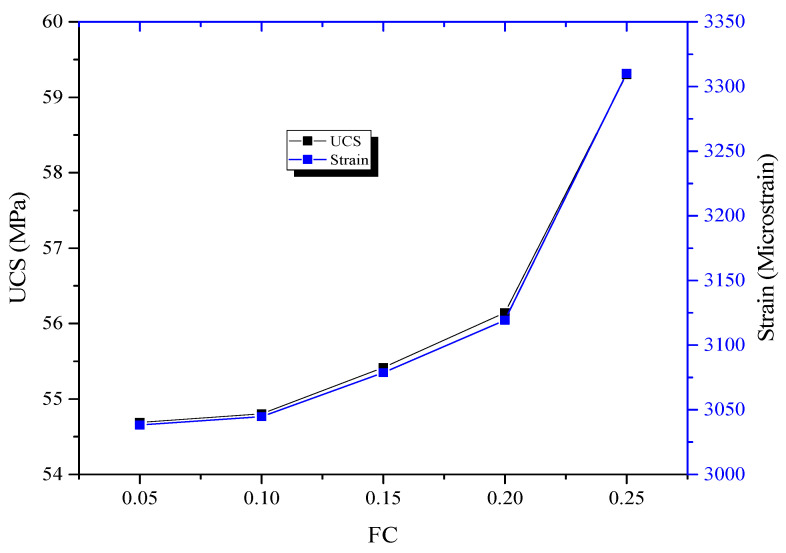
Variation of peak stress and strain with frictional coefficient (FC).

In many testing situations, less attention is given to the influence of FC on rock stress and strain data. In this section, five models were analyzed by varying FC (0.05, 0.1, 0.15, 0.2 and 0.25). Figure 12 indicted high peak stress and strain values when FC was increased. Both peak stress and strain values followed a similar trend. This highlights the importance of lubricating the top and button steel plates while conducting UCS testing to reduce the effect of FC on the experimental results.

## 5. Conclusions

Smart techniques of measuring rock deformation precisely with reasonable accuracy using C-FBG sensors are presented and evaluated. Uniaxial compression tests were performed on samples with sensors attached to the specimen and the machine load cell. C-FBG sensors have been shown to offer the advantages of measuring both radial and axial strain with a single sensor and allowing packaging to ease labour and sensor attachment complications on the sample.

According to the experimental test results, both the FBG_Bare_ and FBG_Pack_ compete with SG and have much higher accuracy than the conventional LVDT. The sensors have demonstrated effectiveness in monitoring the small strain deformational response of rocks with ease, high-resolution, and accuracy. It was found out the stiffness parameters obtain from FBG_Bare_ and FBG_Pack_ nearly the same as that of the SG.

Furthermore, the FEM employed demonstrated the applicability of the concept for measuring correct material properties in the laboratory. The STC analyses revealed the influence of adhesive thickness and packaging material on the sensitivity of both FBG_Bare_ and FBG_Pack_. Even though FBG_Bare_ is more sensitive than FBG_Pack,_ FBG_Pack_ can measure material deformation with reasonable accuracy. Based on the experimental and numerical findings, C-FBG sensors can be utilized to measure strain response in the laboratory.

## Figures and Tables

**Table 1:** Typical specifications of the FBG sensors used for experiments.

S/N	L (mm)	$\mathrm{Wavelength}\;\lambda_{B}\;(\mathrm{nm})$	BW@−3dB (nm)	SLSR (dB)	Reflectivity (%)
1	10	1530.0	0.171	15	95
2	10	1533.7	0.187	13	95
3	10	1537.0	0.184	15	95
4	10	1540.3	0.169	16	95
5	10	1544.0	0.205	14	95
6	10	1547.6	0.184	13	95
7	10	1551.1	0.183	16	95
8	10	1554.5	0.204	15	95
9	10	1558.0	0.199	15	95

L: length of the gratings; λ_B_: central wavelength (CW) corresponding to each grating; SLSR: side lobe suppression ratio: highest secondary peak bigger than 3 dB amplitude within ±3 nm from CW. For standard FBGs SLSR > 15 dB; reflectivity R% = 1–10(T(dB)/10): Measured from transmission spectra.

**Table 2:** UCS and Young’s modulus obtained from all the sensors on each specimen.

S/N	UCS (MPa)	Modulus	FBG_Pack_	FBG_Bare_	SG	LVDT
UC1		*E_t_* (MPa)	30,070	27,300	27,700	20,240
	48.98	*E_av_* (MPa)	29,310	27,630	28,280	20,230
		*E_s_* (MPa)	33,547	33,320	33,456	21,769
UC2		*E_t_* (MPa)	23,820	23,030	22,220	16,770
	45.8	*E_av_* (MPa)	23,790	22,070	21,970	16,840
		*E_s_* (MPa)	23,130	23,131	21,810	15,793
UC10		*E_t_* (MPa)	32,520	31,560	-	28,150
	52.56	*E_av_* (MPa)	32,820	31,890	-	28,080
		*E_s_* (MPa)	35,990	35,892	-	25,022
UC13		*E_t_* (MPa)	29,010	27,560	29,050	22,350
	51.25	*E_av_* (MPa)	28,800	26,880	28,250	21,810
		*E_s_* (MPa)	28,444	26,947	26,148	18,920
UC26		*E_t_* (MPa)	19,300	20,420	19,300	17,100
	32.81	*E_av_* (MPa)	19,135	20,290	19,130	17,350
		*E_s_* (MPa)	20,500	18,222	19,294	13,015
UC31		*E_t_* (MPa)	29,750	28,780	29,870	22,610
	63.27	*E_av_* (MPa)	28,860	27,230	28,050	21,990
		*E_s_* (MPa)	30,170	27,002	28,590	17,416

**Table 3:** Variation of *m* for sensors across the specimens.

Specimen	*m* FBG_Pack_	*m* FBG_Bare_	*m* LVDT
UC1	1.08	0.98	0.73
UC2	1.07	1.04	0.76
UC10	-	-	-
UC13	0.99	0.95	0.77
UC26	1	1.05	0.89
UC31	1.00	0.96	0.76

**Table 4:** Poisson’s ratio of each specimen computed from the sensors.

Specimen	FBG_Pack_	FBG_Bare_	SG
UC1	0.3	0.23	0.21
UC2	0.3	0.24	0.3
UC10	0.27	0.25	
UC13	0.32	0.29	0.32
UC26	0.25	0.23	0.22
UC31	0.21	0.23	0.22

**Table 5:** Properties of model materials.

	Limestone	Steel	Cyano	CFRP	FBGs
*E* (GPa)	29.75	200	1.88	70	73
ν	0.21	0.3	0.2	0.32	0.2
UCS (MPa)	63.27	-	-	-	-
$\sigma_{t}$ (MPa)	6.10	460	23.3	504.3	
κ (GPa)	17.1	166.67	1.04	64.82	43.45
*G* (GPa)	12.3	76.92	0.78	26.52	29.92
ρ (kg/m^3^)	2800	7850	-	1600	2600

**Table 6:** FBG_Pack_ specification for FEM analysis.

Division	Length (mm)	Y (mm)	X (mm)
Class 1	15	0.2	0.05
	15	0.2	0.1
	15	0.2	0.2
Class 2	20	0.2	0.05
	20	0.2	0.1
	20	0.2	0.2
Class 3	30	0.2	0.05
	30	0.2	0.1
	30	0.2	0.2

**Table 7:** Comparison of stiffness parameters of FEM and experimental results.

Specimen	Analysis	*E_t_* (GPa)	*E_av_* (GPa)	*E_s_* (GPa)
UC31	FEM_Rock_	30,560	30,450	30,170
	FEM_FBG_	31,340	31,440	32,192
	FBG_Bare_	28,780	27,230	27,002
	FBG_Pack_	29,750	28,860	30,170
	SG	29,870	28,050	28,590

**Table 8:** Variation of strain-transfer coefficient (STC) with packaging length.

Packaging Length (mm)	STC at x = 0.05 (%)	STC at x = 0.1 (%)	STC at x = 0.2 (%)
15	82.24	81.55	80.33
20	90.14	89.44	88.5
30	93.64	92.87	91.55

## Data Availability

Not applicable.
